# Versatility of the BID Domain: Conserved Function as Type-IV-Secretion-Signal and Secondarily Evolved Effector Functions Within *Bartonella*-Infected Host Cells

**DOI:** 10.3389/fmicb.2019.00921

**Published:** 2019-05-03

**Authors:** Alexander Wagner, Colin Tittes, Christoph Dehio

**Affiliations:** Biozentrum, University of Basel, Basel, Switzerland

**Keywords:** Type-IV-secretion, VirB/VirD4, Bartonella, α-proteobacteria, Bartonella effector proteins, relaxases, BID domain, pathogenesis

## Abstract

*Bartonella* spp. are facultative intracellular pathogens that infect a wide range of mammalian hosts including humans. In order to subvert cellular functions and the innate immune response of their hosts, these pathogens utilize a VirB/VirD4 type-IV-secretion (T4S) system to translocate *Bartonella* effector proteins (Beps) into host cells. Crucial for this process is the Bep intracellular delivery (BID) domain that together with a C-terminal stretch of positively charged residues constitutes a bipartite T4S signal. This function in T4S is evolutionarily conserved with BID domains present in bacterial toxins and relaxases. Strikingly, some BID domains of Beps have evolved secondary functions to modulate host cell and innate immune pathways in favor of *Bartonella* infection. For instance, BID domains mediate F-actin-dependent bacterial internalization, inhibition of apoptosis, or modulate cell migration. Recently, crystal structures of three BID domains from different Beps have been solved, revealing a conserved fold formed by a four-helix bundle topped with a hook. While the conserved BID domain fold might preserve its genuine role in T4S, the highly variable surfaces characteristic for BID domains may facilitate secondary functions. In this review, we summarize our current knowledge on evolutionary and structural traits as well as functional aspects of the BID domain with regard to T4S and pathogenesis.

## Introduction

Type-IV-secretion (T4S) systems are multiprotein complexes embedded in the cell envelope of Gram-negative and Gram-positive bacteria and some archaea (Berge et al., 2017). They represent versatile nanomachines that fulfill diverse functions including (1) contact-dependent transfer of DNA (conjugation), (2) contact-dependent transfer of bacterial effector proteins into eukaryotic host cells, (3) contact-dependent toxin delivery into bacterial cells, (4) secretion of DNA into the extracellular milieu, and (5) uptake of DNA from the environment (Waksman, 2019). T4S systems can be, based on their architectural complexity, categorized into two classes: T4AS (12 subunits) and T4BS (>25 subunits) systems (Waksman, 2019). Based on the paradigmatic *Agrobacterium tumefaciens* VirB/VirD4 T4AS system, the subunits are named VirB1–11 and VirD4 (Li and Christie, 2018). VirB2–11 are essential for the assembly of the T4S system machinery and substrate translocation. The membrane-bound ATPase VirD4—also known as the T4S coupling protein (T4CP)—is crucial for the recognition of T4S substrates prior to translocation.

**Figure 1:**
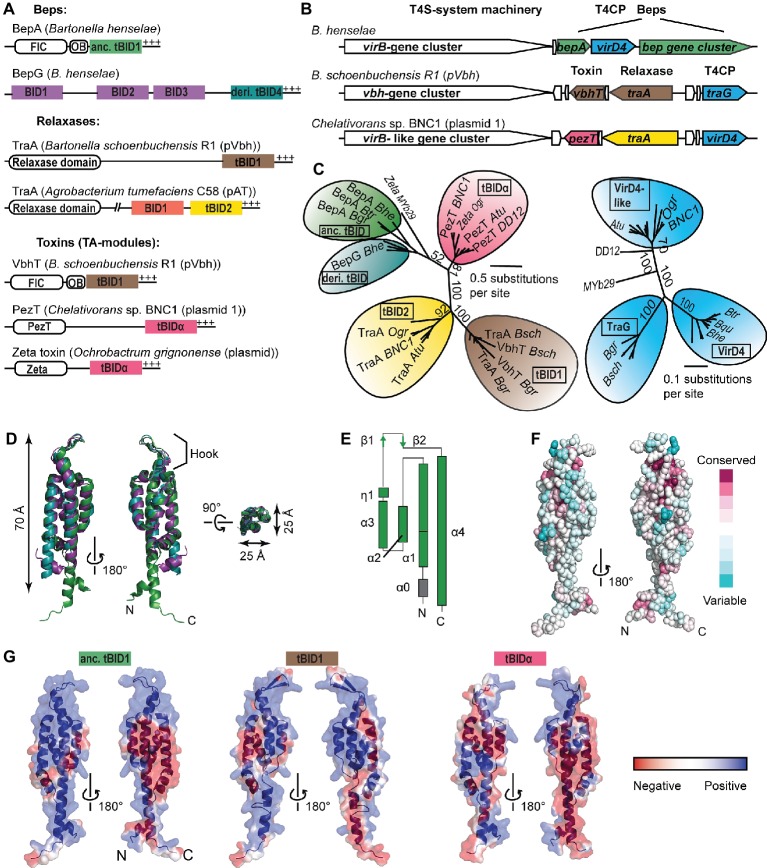
Classification and structural features of BID domains. **(A)** Domain architecture of BID domain-containing type-IV-secretion (T4S) substrates found within α-proteobacteria. N-terminal, catalytic domains (FIC, relaxase domain, PezT, Zeta) and the OB (oligonucleotide binding) fold are displayed as white rectangles, respectively. BIDx and tBIDx domains are colored based on the subclass. +++, positively charged tail; anc., ancestral; deri., derived. **(B)** Synteny of representative BID domain-containing T4S substrate genes and adjacent *virB/virD4-like* T4S-system locus and genes involved in plasmid conjugation (the latter two are represented in white). The color code of BID domain-encoding genes is the same as of the respective BID domains displayed in **(A)**. Homologs of T4S coupling protein (T4CP) are depicted in blue. **(C)** Simplified neighbor-joining distance-based tree representations of the multiple sequence alignment of (left) terminal BIDs [tBIDs, color code as in **(A)**] and (right) the soluble domains of VirD4-like T4CPs that are associated with the tBIDs. Highlighted are representative proteins (per subfamily) encoded by: *B. grahamii (Bgr), B. tribocorum (Btr), B. henselae (Bhe), B. schoenbuchensis pVbh (Bsch), Agrobacterium tumefaciens* C58 pAT (*Atu), Chelativorans* sp. BNC1 plasmid 1 (BNC1), *Ochrobactrum grignonense* plasmid (*Ogr), Ochrobactrum sp.* MYb29 (MYb29), and *Shinella sp*. DD12 (DD12). **(D)** The similarity and compact nature of the BID fold is highlighted through superposition of the three solved BID domains: *Bro*Bep6_tBID1 (green; PDB: 4YK1), *Bcl*Bep9_tBID1 (cyan; PDB: 4YK2), and *Bhe*BepE_BID1 (purple; PDB: 4YK3). **(E)** Topology representation of *Bro*Bep6_tBID1. **(D,E)** are redrawn from Stanger et al. (2017) to adapt the color code to the remaining figure. **(F)** Residue conservation is rather low among BID domains of Beps. Depicted is the overall conservation score of BID domains mapped on the surface representation of *Bro*Bep6_tBID1. The color code is based on the ConSurf color scale (Ashkenazy et al., 2016). **(G)** Electrostatic potential mapped on the surfaces of experimentally determined (anc. tBID1 of *Bro*Bep6) and modeled (tBID1 of VbhT from *Bsch* and tBIDα from PezT from BNC1) tBID domains. Protein backbones are depicted as cartoon representation. The color code highlighting the electrostatic potential ranges from red (negative) to blue (positive).

The majority of T4S substrates possess a non-cleavable T4S signal at their C-termini consisting of only a few positively charged or hydrophobic residues (Christie et al., 2014). However, some T4S signals form a larger structural scaffold, as for instance, the globular TSA domain of conjugative relaxase TraI encoded by plasmid R1 (Redzej et al., 2013). Another example constitutes the approximately 100-aa-long BID (Bep intracellular delivery) domain that together with a short positively charged C-terminal tail forms a bipartite T4S signal proposed to interact with the T4CP (Schulein et al., 2005; Stanger et al., 2017). The BID domain is present in α-proteobacterial toxins, relaxases, and Beps (*Bartonella* effector proteins) (Figure 1A), the latter representing numerous host cell-targeted effectors of pathogenic *Bartonella* species (Wagner and Dehio, 2019). The vast majority of BID domain-containing T4S substrates are genetically linked to a T4S system that resembles the canonical *A. tumefaciens* VirB/VirD4 T4S system (Figure 1B). VirD4-T4CPs associated with BID domain-containing T4S substrates form a monophyletic group among T4CP subtypes, indicating a coevolutionary trajectory to maintain interaction of this sublineage with BID domains (Schulein et al., 2005). Multiple studies with the model pathogen *Bartonella henselae* (*Bhe*) showed that Beps are translocated *via* a VirB/VirD4 T4S system into various host cell types to modulate diverse cellular and innate immune functions allowing *Bartonella* to spread and establish long-lasting hemotrophic infections in its mammalian host (Schulein et al., 2005; Schmid et al., 2006; Truttmann et al., 2011a; Okujava et al., 2014).

Beps are multi-domain proteins. A majority of Beps possess an N-terminal FIC (Filamentation induced by cAMP) domain that confers posttranslational modifications, a central connecting OB (oligonucleotide binding) fold, and a C-terminal BID domain (Engel et al., 2011; Harms et al., 2017b). A previous genome analysis revealed that 70% of all Beps and the interbacterial toxin VbhT (present on conjugative plasmid pVbh encoding the Vbh (VirB homologous) T4S system in certain *Bartonella* spp.) display the canonical FIC-OB-BID architecture (Engel et al., 2011). Furthermore, all Bep repertoires present in the *Bartonella* genus harbor Beps with a derived domain composition, which likely evolved from a single primordial FIC-OB-BID ancestor *via* repeated gene duplication and diversification events. The diversification of Bep repertoires occurred independently in three distinct *Bartonella* lineages, resulting in Bep197–234 in *B. ancashensis* of lineage 1 (L-1), Bep1–10 in lineage 3 (L-3), and BepA-I in the most species-rich lineage 4 (L-4) (Harms et al., 2017). Instead of a FIC domain, the derived Beps possess tandem-repeated tyrosine phosphorylation (pY) motifs that are phosphorylated by endogenous host cell kinases, and/or additional BID domains (Wagner and Dehio, 2019). While the original function as T4S signal is preserved among C-terminal BID domains, some BID domains have secondarily evolved effector functions within host cells. These functions include inhibition of apoptosis, bacterial uptake *via* rearrangement of the F-actin cytoskeleton, and modulation of cell migration of infected host cells (Truttmann et al., 2011a; Pulliainen et al., 2012; Okujava et al., 2014).

In this review, we will focus on the evolution and classification of BID domains based on their presence in different T4S substrates. We will furthermore discuss structural and functional aspects of the BID domain with regard to T4S and the subversion of host cell function.

## Classification And Structural Features Of BID Domains

### Classification of BID Domains

BID domains display high sequence variability. Their classification is mainly based on their position within the multi-domain relaxases and Beps (Figure 1A; Stanger et al., 2017). C-terminal BID domains that serve as part of the T4S signal are designated as tBIDx and nonterminal BID domains as BIDx, with “x” representing a number indicating whether this BID domain is the first, second, third, or fourth BID domain counted from the N-terminus (Figure 1A). In the Beps, tBIDs have been further subclassified into ancestral tBIDs found in the canonical FIC-OB-BID architecture and derived tBIDs of pY- and multi-BID domain-containing Beps (Stanger et al., 2017).

A recent study reported the presence of BID domains fused to toxin domains (PezT and Zeta) of toxin/antitoxin modules (Harms et al., 2017a). These PezT/Zeta-BID proteins are encoded by conjugative plasmids that are prevalent in various α-proteobacterial genera such as *Agrobacterium*, *Chelativorans*, *Ochrobactrum*, and *Sinorhizobium*. Although it is not clear whether these PezT/Zeta-BID proteins are translocated through a T4S system, their genetic conservation to a *virB/virD4*-like T4S system locus suggests that these proteins are *bona fide* T4S substrates (Figure 1B; Harms et al., 2017a; Wagner and Dehio, 2019). Therefore, we extend the classification of BID domains by introducing tBIDα for those present in toxins found in various α-proteobacterial species. In a phylogenetic tree, tBIDα domains form a monophyletic group similar to tBID2 domains of α-proteobacterial relaxases, tBID1 domains of *Bartonella* TraA-relaxases/VbhTs, and the ancient and derived tBID domains found in Beps, confirming that the tBIDα domains form a new class of BID domains (Figure 1C, left). Interestingly, tBIDα domains are more closely related to the tBID domains of Beps than to tBID2 domains of α-proteobacterial relaxases, even though PezT/Zeta-BID proteins are encoded adjacent to the latter (Figure 1B). Thus, tBIDα domains are likely not the result of gene duplication and reshuffling events of relaxase tBID1/2 domains as proposed for the tBID1 domain of VbhT that is virtually identical to the tBID1 domains of *Bartonella* TraA relaxases (Harms et al., 2017a).

The coevolutionary trajectory of tBID domains with their cognate T4CP is evident for tBID1 domains (TraA/VbhT) and TraG and for ancestral/derived tBIDs (Beps) with VirD4 (Figure 1C). In contrast, tBID2 (α-proteobacterial relaxases)- and tBIDα (PezT/Zeta-BID)-containing proteins supposedly interact with the same VirD4-like T4CP (Figure 1C, right), although these tBID domains are as distantly related to each other as tBID1 domains and ancestral/derived tBID domains (Figure 1C, left). It is worth mentioning that translocation of tBID-containing substrates through heterologous VirB/VirD4 T4S systems has been experimentally proven in multiple instances (e.g., Schulein et al., 2005; Harms et al., 2017a). For instance, the tBID2 domain of α-proteobacterial relaxase TraA (encoded on *Atu* pAT) fused to Cre-recombinase (Cre) translocates through the *Bhe* VirB/VirD4 T4S system with similar efficiency as Cre-tBID-fusions of different *Bhe*-Beps (Schulein et al., 2005). We therefore believe that coevolution of tBID domains with their BID-associated VirD4-T4CPs has not yet led to the establishment of discrete specificities in recognition – despite the remarkable sequence variability of BID domains.

### Structural Features of BID Domains

Recently, the crystal structures of BID domains representative for three different classes have been solved, including an ancestral tBID1 domain of *B. rochalimae* Bep6, a derived tBID1 domain of *B. clarridgeiae* Bep9, and a derived BID1 domain of *Bhe*-BepE (Stanger et al., 2017). All three BID domains are folded to a rigid, antiparallel four-helix bundle topped with a hook that accommodates a position opposite to both termini (Figures 1D,E). The shape of the three BID domains is elongated with a length of approximately 70 Å and a diameter of 25 Å, suggesting that this conserved fold might be crucial for the primary secretion signal function of the BID domain in T4S (Stanger et al., 2017). It is tempting to speculate that the hook might play a role in the interaction with the T4CP and/or other components of the VirB/VirD4 T4S machinery. Helix-1 (α1) and helix-4 (α4) can either be straight or kinked (Figure 1D). This conformational variability of BID domains at their extremities might be the result of different domain compositions in the remaining part of the protein and/or facilitate novel interactions with host target proteins. Interestingly, the T4S activity of a Cre-tBID1-fusion lacking α1 was reduced to 30% compared to full-length Cre-tBID1, suggesting an important role of α1 in translocation (Schulein et al., 2005).

While the hydrophobic core of BID domains is conserved, their surfaces are highly variable. The plasticity of BID domains is highlighted by the fact that BID domains of relaxases and Beps display on average 14% similarity (Stanger et al., 2017). Sequence similarity is also rather low within the various BID domain classes (Figure 1F), with the highest degree of similarity observed within tBID1 domains of *Bartonella* TraA and VbhT, respectively. In general, tBIDx subclasses display a higher degree of conservation than BIDx classes, which might be due to a relieved selection pressure of BIDx domains to interact with the T4CP. Although the surface composition among BID domains is poorly conserved, their surface charge distribution seems to be rather consistent, displaying two highly positively charged areas separated by a negatively charged patch (Stanger et al., 2017). This mainly positive charge distribution suggests that BID domains likely interact with a negatively charged surface on the interaction partner. Furthermore, slight differences in the charge distribution between BID domain classes and even among closely related BID domain orthologs can be observed (Figure 1G), suggesting subtle functional differences with respect to T4S efficiency (tBIDx) and effector function (BIDx/tBIDx).

Summarizing, we believe that the conserved rigid fold of the BID domain facilitates its role as T4S signal, whereas the highly variable surface might enable the evolution of secondary functions within host cells.

## BID Domain-Mediated Host Cell Modulations

**Figure 2:**
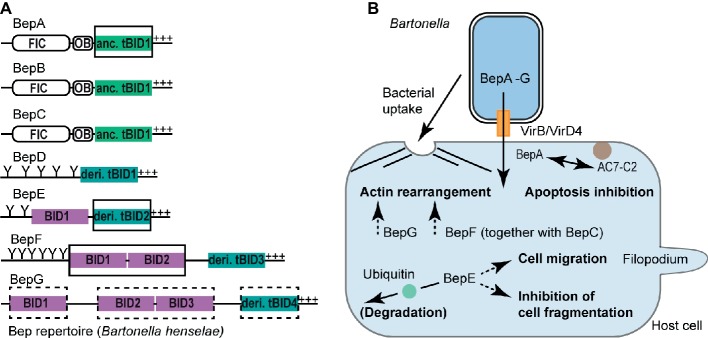
BID domain-mediated host cell modulations. **(A)** Representative Bep repertoire of the model organism *Bartonella henselae* highlighting various classes of BID domains. BID domains with experimentally proven or suspected host-modulating function are highlighted with a full or dashed rectangle, respectively. OB, Oligonucleotide binding fold; Y, Tyrosine phosphorylation motif; +++, positively charged tail; anc., ancestral; deri., derived. **(B)** Schematic representation of BID-mediated host cell modulations. Following the VirB/VirD4 T4S system-mediated translocation into host cells, distinct BID domains [highlighted in **(A)**] alter host cell signaling processes allowing *Bartonella* to survive and propagate within the mammalian host. Host cells can counteract Bep-mediated functions by degrading the effectors following ubiquitination of their BID domains as shown for the BID domains of *Bqu*-BepE. Confirmed BID-target interactions are displayed with a continuous arrow, whereas dashed arrows indicate BID-mediated host cell modulations for which the target protein(s) are currently unknown. AC7-C2, catalytic subunit C2 of human adenylyl cyclase isoform 7 (AC7).

The remarkable degree of host adaptation of pathogenic *Bartonella* spp. to their mammalian hosts has been attributed to a large extent to the VirB/VirD4 T4S system and its translocated Beps (Siamer and Dehio, 2015; Dehio and Tsolis, 2017; Wagner and Dehio, 2019). Most of the Bep-mediated host cell modulations are BID domain dependent and include apoptosis inhibition, F-actin rearrangements, and host cell migration. BID domain-mediated phenotypes are best understood on the molecular and cellular level for human pathogenic *Bhe* and *B. quintana (Bqu*). Both species belong to L-4 and thus their Beps are designated with a letter code (BepA, BepB, and so on, Figure 2A).

### BepA-tBID1 Mediates Apoptosis Inhibition in a Host-Specific Manner

*Bhe* and *Bqu* enhance the proliferation of human endothelial cells (ECs) by inhibiting apoptosis (Kirby and Nekorchuk, 2002; Schmid et al., 2006; Pulliainen et al., 2012). The antiapoptotic activity was assigned to the ancestral tBID1 domain of *Bhe*-BepA and *Bqu*-BepA.2, respectively (Figure 2B). *Bhe*-BepA-tBID1 physically interacts with the catalytic subunit C2 of human adenylyl cyclase isoform 7 (AC7) to potentiate cAMP production (Pulliainen et al., 2012). AC7 is a plasma membrane-bound enzyme, which requires activation and association of its two catalytic subunits C1 and C2 to convert ATP into cAMP (Sadana and Dessauer, 2009). *Bhe*-BepA-tBID1 potentiates the cAMP-triggering effect of GTP-bound Gα_S_ in a physiological context and in a pharmacological context together with the plant-derived drug forskolin, which intercalates C1 and C2 into a catalytically active form (Pulliainen et al., 2012). Thus, it is plausible to assume that *Bhe*-BepA-tBID1 allosterically enhances C1 and C2 subunit association in order to elevate cAMP production. The molecular restraints of the *Bhe*-BepA-tBID1 interaction with C2 (AC7) are currently unknown. However, a *Bhe*-BepA construct consisting only of helix-4 of the ancestral tBID1 domain and the adjacent C-terminal stretch did not inhibit apoptosis, suggesting that helix-4 alone is not sufficient for the antiapoptotic activity mediated by *Bhe*-BepA-BID (Schmid et al., 2006).

Interestingly, neither the BepA paralogs BepB or BepC of *Bhe* nor the BepA ortholog from the rat-associated *B. tribocorum (Btr*) displayed antiapoptotic activity (Schmid et al., 2006). The latter is in line with previous observations that only L-4 species associated with significant clinical manifestations in humans (e.g., *Bhe* and *Bqu*) possess antiapoptotic activity toward human ECs (Kirby and Nekorchuk, 2002).

### Multi-BID Domain-Containing Effectors BepG and BepF Trigger Invasome Formation

*Bhe* cells are internalized into ECs either individually *via* endocytosis or as bacterial aggregates in the course of the formation of a unique cellular structure, the invasome (Dehio et al., 1997). Invasome formation is a multistep process, that, following bacterial accumulation at the EC surface, involves F-actin rearrangements and stress fiber formation underneath the engulfed bacterial aggregate (Dehio et al., 1997). Furthermore, in contrast to the T4S system-independent endocytic uptake of *Bhe* into ECs, invasome formation is strictly VirB/VirD4 dependent (Schmid et al., 2004) and is redundantly triggered by either BepG or through the combined action of BepC and BepF (Figure 2B; Rhomberg et al., 2009; Truttmann et al., 2011b).

BepG of *Bhe* consists solely of four BID domains that are connected *via* short linker sequences. Hence, BepG likely promotes invasome formation *via* at least one of these four BID domains through interaction with yet unknown host target protein(s) (Rhomberg et al., 2009). Similarly, invasome formation mediated by BepF (together with BepC) is triggered by its two nonterminal BID domains BID1 and BID2, but not by its derived tBID3 domain (Truttmann et al., 2011a). Interestingly, BID2 and tBID3 are more similar to each other than to BID1. The overall low sequence conservation of these three BID domains allows no conclusion on why BID1 and BID2 contribute to invasome formation, but tBID3 is negligible in this process. We speculate that, besides certain, non-conserved residues at the surface of BID domains, also the relative position of BID domains within multi-domain architectures may be critical for effector function.

### BepE-BID Domains Antagonize Cell Fragmentation and Promote Host Cell Migration

*Bartonella* spp. supposedly infect dendritic cells at the dermal site of inoculation and exploit these migratory cells as Trojan horses in order to reach the bloodstream, where bacteria infect, replicate, and persist within erythrocytes (Scherer et al., 1993; Schulein et al., 2001; Okujava et al., 2014; Vieira-Damiani et al., 2016). *In vivo* dissemination into the bloodstream is VirB/VirD4 dependent and relies on the function of BepE (Figure 2B; Okujava et al., 2014). *Bhe*-BepE is a pY-containing Bep that contains two BID domains (Figure 2A). Although *Bhe*-BepE interacts *via* its pY motifs with several host cell signaling proteins (Selbach et al., 2009), the dissemination of the bacteria into the bloodstream is exclusively dependent on the BID domains. *In vitro*, the derived tBID2 domain of *Bhe*-BepE interferes with a deleterious cell fragmentation phenotype triggered by other Beps (Okujava et al., 2014). Inhibition of the cell fragmentation phenotype is not only restricted to *Bhe*-BepE but appears to be a conserved function among BepE homologs including *Bqu*-BepE and *Btr*-BepE (Okujava et al., 2014).

Besides the cytoprotective effect against other Beps, translocated *Bhe*-BepE promotes the migratory capability of dendritic cells (Okujava et al., 2014). It needs to be determined whether this effect is mediated by the BID domains of BepE or by its pY motifs. However, it is conceivable that the promotion of cell migration is BID dependent, as BID domains of *Bhe*-BepE are sufficient to enable *Bartonella* cells to reach the bloodstream *in vivo* (Okujava et al., 2014).

A recent study showed that the tandemly repeated BID domains of *Bqu*-BepE but not of *Bhe*-BepE become ubiquitinated within host cells followed by proteasomal degradation (Wang et al., 2018). Whether or not this ubiquitination plays a role in effector function of *Bqu*-BepE needs to be addressed in future research.

## Concluding Remarks

The BID domain constitutes together with a positively charged C-terminal tail a T4S-signal that is crucial for the interbacterial transfer of relaxases (alongside plasmid conjugation) and protein toxins by various α-proteobacteria and for the interkingdom transfer of effectors by pathogenic *Bartonella* spp. Besides its genuine role in T4S, several BID domains have adopted secondary functions within host cells such as apoptosis inhibition and F-actin rearrangements. On the basis of the recently solved BID domain structures, their conserved fold may play a crucial role in T4S, whereas the plasticity of the surfaces seems to have facilitated novel interaction areas with host target proteins (Stanger et al., 2017). Future structure/function-related studies should aim to systematically determine key residues of BID domain interactions with the T4CP and/or other T4S system components and with host target proteins. Regarding the latter, comparative analyses of BID domain-host protein interactions evolved in specific *Bartonella*-mammal pairs will enable us to address the role of Beps in host adaptation.

Independent studies showed that T4S substrates are translocated to the recipient cytosol in an unfolded state (Amyot et al., 2013; Trokter and Waksman, 2018). Due to its structural similarity with the intramolecular chaperone IpaD of type-III-secretion systems, the BID domain might similarly act as an unfoldase to prime toxins, relaxases, and Beps for translocation (Stanger et al., 2017). Future work may address the question if BID domains indeed function as chaperones prior to and after T4S. Furthermore, using multidisciplinary approaches such as X-ray crystallography and cryo-electron tomography, future efforts could pave the way for elucidating long-lasting questions regarding the T4S pathway(s) of relaxases and effectors by using BID domain-containing T4S substrates.

## Author Contributions

AW and CT designed the figures. AW, CT, and CD wrote the manuscript.

### Conflict of Interest Statement

The authors declare that the research was conducted in the absence of any commercial or financial relationships that could be construed as a potential conflict of interest.
